# Endometriosis: current challenges in modeling a multifactorial disease of unknown etiology

**DOI:** 10.1186/s12967-020-02471-0

**Published:** 2020-08-12

**Authors:** Helena Malvezzi, Eliana Blini Marengo, Sérgio Podgaec, Carla de Azevedo Piccinato

**Affiliations:** 1 Hospital Israelita Albert Einstein, São Paulo, SP 05652-900 Brazil; 2 Instituto Butanta- EstabilidadeBiotech Quality Control, São Paulo, SP 05503-900 Brazil

**Keywords:** Endometriosis, Animal model, Drug efficacy, Organ-on-chip, Cell culture, Artificial intelligence

## Abstract

Endometriosis is a chronic inflammatory hormone-dependent condition associated with pelvic pain and infertility, characterized by the growth of ectopic endometrium outside the uterus. Given its still unknown etiology, treatments usually aim at diminishing pain and/or achieving pregnancy. Despite some progress in defining mode-of-action for drug development, the lack of reliable animal models indicates that novel approaches are required. The difficulties inherent to modeling endometriosis are related to its multifactorial nature, a condition that hinders the recreation of its pathology and the identification of clinically relevant metrics to assess drug efficacy. In this review, we report and comment endometriosis models and how they have led to new therapies. We envision a roadmap for endometriosis research, integrating Artificial Intelligence, three-dimensional cultures and organ-on-chip models as ways to achieve better understanding of physiopathological features and better tailored effective treatments.

## Background: setting and disease

Endometriosis is a reproductive age-associated disease [1, 2] that has become the target of intense investigation, as indicated by the increasing number of scientific papers published. In the last 10 years, indeed, more than 75% of endometriosis-related papers, appeared during that period according to Web of Science data. This surge of information directed to both laypersons and healthcare professionals improved the identification of symptoms, augmented the odds of correct diagnosis as well as the awareness of available medical treatment [3].

**Fig. 1:**
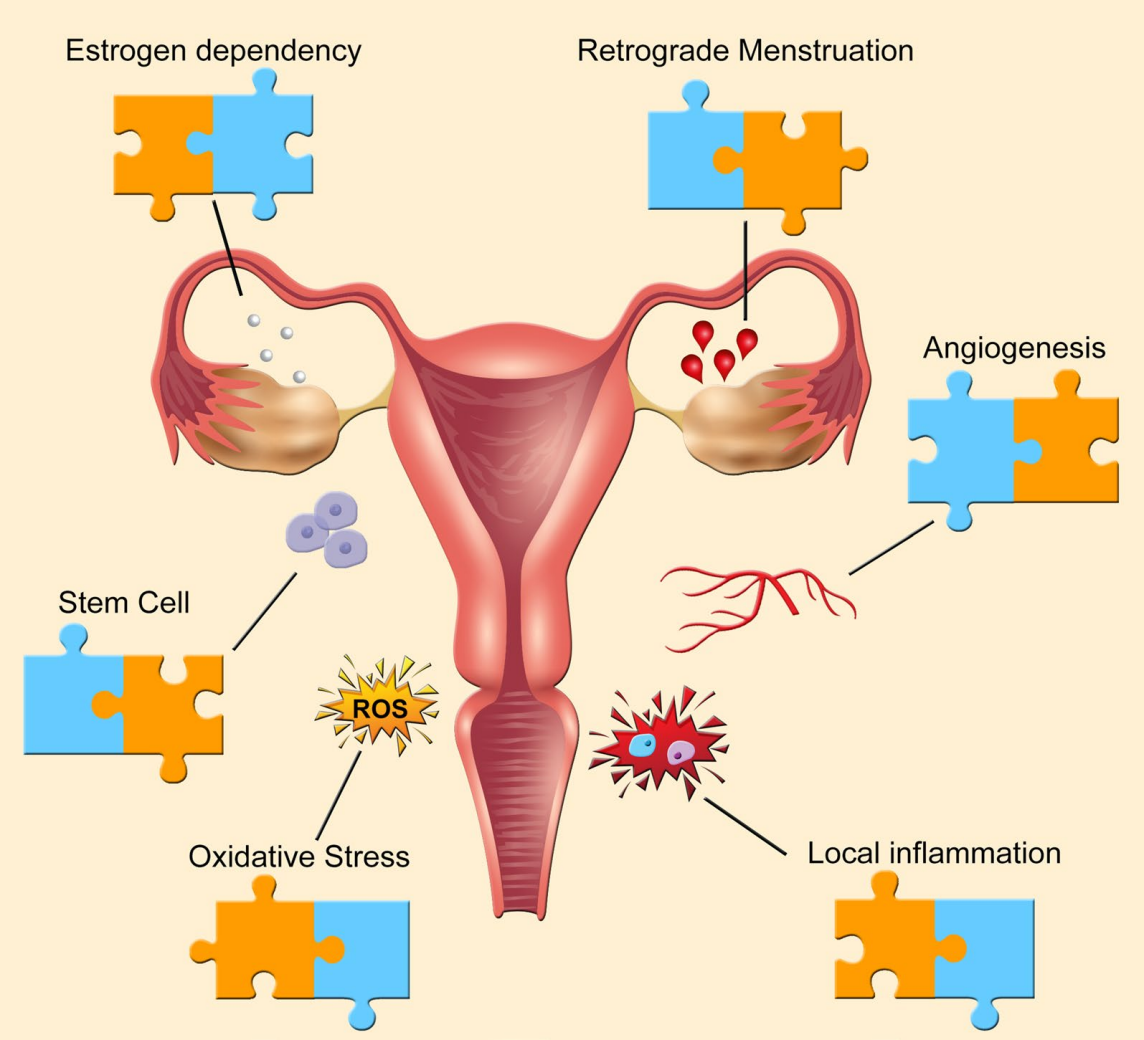
Endometriosis characteristics. Legend: Endometriosis main characteristics and contributions to its knowledge—shown as puzzle pieces—coming from studies using animal (blue pieces) or human-based models (orange pieces)

Worldwide epidemiological studies show a mean prevalence of 10% of endometriosis in the pre-menopausal population [4], with annual incidences in specific populations varying from 0.112% [5] to 0.72% [6]. The difficulties to reach the diagnosis comprise the need for clinical and surgical expertise to evaluate correctly the clinical symptoms and to detect the presence of ectopic endometrial implants (so-called lesions) in the peritoneal cavity and on pelvic organs [7]. Although imaging methods such as transvaginal ultrasonography with bowel preparation and magnetic resonance are common diagnostic tools, the gold standard diagnostic method for endometriosis is still the histopathological analysis of lesions collected during laparoscopic surgery [8]. The etiopathogenesis of endometriosis is not known. However, there are theories on the origin of endometriotic lesions in the peritoneal cavity. It is proposed by some investigators that stem cells originating the lesions are already there in the peritoneal cavity whereas others propose that endometrium cells are seeded there by retrograde menstruation. The pathophysiology, however, is strongly influenced by other factors such as genetic predisposition and hormonal factors such as resistance to progesterone, estrogen dependence; inflammation, angiogenesis, and vascularization processes, oxidative stress, resistance to apoptosis and immunological factors are also involved to various degrees in lesion development (Fig. 1).

It is generally accepted that the lesions found in different pelvic sites are the main cause of all clinical findings in endometriosis. However, many women have lesions while being asymptomatic, and such lesions are found during abdominal surgery done for reasons other than endometriosis. The prevalence of asymptomatic cases among studies is highly variable ranging from 6% [9] up to 43.3% [10, 11]. The common clinical symptoms associated with endometriosis vary from mild to severe pain and/or infertility. The pain observed in 30 to 80% of the cases can manifest itself as dysmenorrhea, acyclic pelvic pain, deep dyspareunia, dysuria and dyschezia [12]. Infertility is found in 30 to 40% of the cases [6]. Other symptoms are fatigue, diarrhea, constipation, bloating or nausea, mainly during menstrual periods, and pain-related to mental health problems such as somatization or depression, heightened sensitivity and anxiety are also seen in endometriosis patients when compared to control groups [13].

The treatment for endometriosis usually aims at ameliorating symptoms, mainly pain, and/or achieving pregnancy in infertile patients [14]. Early understanding of the disease has come from experimental studies in animals [15] while advances in the therapy of endometriosis have come from observation of symptomatic patients during surgery [16], from research on patients’ data and from in vitro cell cultures [17].

## The challenge of modeling endometriosis

Currently, the main approaches to investigate endometriosis are human-based either in vivo or in vitro, using cells or tissue samples. There are also experimental in vivo animal models [18]. The first type encompasses clinical trials, patients’ observational and association studies. Experimental in vitro studies include histopathological comparative experiments, as well as tissue fragments, cells and fluids obtained from resected lesions or aspiration biopsies, in general called patient-derived tissue or fluid. In vivo animal models are still required to test drug candidates that affect these processes and to support preclinical trial testing.

The development of in vitro or in vivo models that recreate or exhibit the main characteristics of endometriosis is a challenging task for several reasons. Firstly, it is not known how the disease starts and how it persists. Secondly, endometriosis comprises distinctive disease features such as ovarian endometrioma, and peritoneal as well as deep infiltrative lesions. Lastly, endometriosis has not a single pathophysiological process on which basis it could be modeled. Indeed, being a multifactorial and complex disease, endometriosis has also been associated to environmental [19], genetic [20], immunological factors [21] and hormonal changes (such as estrogen-dependency [22] and progesterone resistance [23]).

The difficulties to evaluate and model the onset of endometriosis stem because it is not clear when it actually starts. It is accepted and reported that many women had already had the disease for up to 12 years before the appearance of symptoms [24]. The main mechanism put forward to explain the beginning of that disease is based on retrospective epidemiological studies [25]. They show that retrograde menstruation [16] is associated with endometrial implants attached to the peritoneal cavity, which would develop into endometrial lesions. However, almost all reproductive-age women (90%) have retrograde menstruation to the pelvic cavity, but only 10% are actually affected by endometriosis. This suggests that other associated factors are needed, besides retrograde menstruation, for the onset and progression of the disease in the peritoneal cavity [26].

Thus, the main current challenge to develop research models for the investigation of endometriosis goes beyond the creation of models that fully recapitulate the multitude of factors affecting this disease; that challenge requires the definition of measurable and clinically translatable effective endpoints.

Generally speaking, there is still a gap in the development of adequate disease models to study essential biological processes [27], as shown by the fact that between 50 and 70% of drugs that reach phase II and III in clinical trials fail to demonstrate effectiveness [28, 29]. Particularly, an ideal model to investigate endometriosis should incorporate relevant disease characteristics, such as the same cellular pathways and the clinical behavior observed in patients.

Until now, the most common approach to this problem involved diverse animal models whose pathophysiological processes are claimed to be somehow similar to the human ones [30]. This idea relies on the concept that the use of animal models facilitates the analysis of integrative and complex events that occur in vivo. Some may claim that the biological systems in all species are essentially a network of cellular and molecular mediators working together for the organism’s survival.

Rodents and non-human primates are the most common animal-based models in endometriosis research with advantages and disadvantages. Rodents, such as rats, mice, and hamsters have been used to investigate basic mechanisms and the development of new drugs. They are easy to handle and relatively inexpensive animals to work with; however, endometriosis does not develop naturally in rodents, possibly because the endometrium is not shed during the estrous cycle. To work with this model, homologous or heterologous uterine tissue has to be surgically introduced in the peritoneal cavity of these animals [31].

Conversely, endometriosis develops spontaneously in non-human primate models that have a natural menstrual cycle, such as rhesus monkeys and baboons [32]. Similarly to women, the duration of the menstrual cycle in female Rhesus (*Macaca mulatta*), cynomolgus *(Macaca fascicularis)* and pigtailed macaques (*Macaca nemestrina)*) is around 28 days [33]. These animals also have retrograde menstruation and their reproductive anatomy (uterus, fallopian tubes and ovaries morphology) and endocrine influences are similar to humans [34]. Although non-human primates would be interesting models to study endometriosis, they are captivity-sensitive animals and costly to maintain. In addition, spontaneous endometriosis develops at considerable low frequencies, which limits primates’ use for research purposes. Moreover, public opposition to non-human primate research has been rising [35] and stricter recommendations exist for future work with non-human primates are being designed. The latest report from the Scientific Committee on Health Environmental and Emerging Risks (SCHEER) on the need for non-human primates in biomedical research, production and testing of products and devices, updated in 2017, proposes 23 recommendations for future work with non-human primates.

Some of those recommendations are requirements already listed in the Europe Union Directive 2010/63, and have been merged into the committees’ series of recommendations in order to amplify their importance and to encourage full and rapid implementation, such as: to check, case by case, the need of using non-human primates; to provide solid harm-benefit assessment of using non-human primates; and to identify circumstances in which to avoid using non-human primates [36].

Other less representative models used in endometriosis studies are chicken, rabbit, sheep and cow [37]. The studies using these models frequently focus on a particular physiopathological aspect, which can be emulated. Chickens have been used for disease mechanism studies, as for instance, a chicken embryo chorioallantoic membrane model was used to show the invasiveness potential of epithelial and stromal endometriosis cells [40] and the role of angiogenesis in lesions formation [38]. In turn, rabbits and cows have been used to model key points of endometriosis-associated infertility. The understanding of reduced fertility with the increase of prostaglandins was observed first in studies using rabbits [41]. In vitro oocyte maturation studies, using bovine oocytes and follicular fluid collected from women with endometriosis, showed damage to the meiotic spindle, probably caused by elevated oxidative stress [39, 42].

Even though some contribution to advancing the knowledge of endometriosis physiopathology originated from animal research, none of the reported animal models has yet led to successful novel therapies [43, 44]. In contrast, patient tissue or fluid-derived in vitro models as well as other human-based models, such as patient tissue and observation studies, have given substantial knowledge to the development of possible therapy targets, which could be tested in human-based clinical trials. In vitro models present several advantages over animal models, namely the easy access to target cells enabling identification of critical cellular and molecular contributors to the disease. Although not contemplating the complete biological system, in vitro models enable high-throughput screening for therapeutic compounds, with overall lower ethical issues and costs. Important findings on the physiopathology of endometriosis have come from classic in vitro cultures of endometriosis-derived stromal [45] or epithelial cells [46]; more recently three-dimensional (3D) cultures [47] also came into use with several advantages.

## Animal models

### Contribution of studies with non-human primates to endometriosis research

Much of the knowledge of pathophysiological processes such as tissue attachment [18], endometriosis-related pain mediators [12], hormonal dependency, progesterone resistance, angiogenesis, oxidative stress and inflammation has evolved from research on non-human primate models done in the past [48] and also from non-animal research [25]. The main reason for conducting research on human disease mechanisms in animal models is the simulation of the disease and, eventually, the translation of findings to humans. Although most of animal model studies in general, including non-human primate models, have been used to understand the mechanism of action of approved drugs on endometriotic lesions and on the treatment of other symptoms, they have also failed to provide convincing translatable results related to the disease mechanisms, toxicity of compounds, teratogenic effects, correct dosage and drugs delivery routes [49]. Many studies using non-human primates were repeated many times to the point that ethical reasons emerged to restrict the number of animals in investigation.

An experiment in baboons with endometriosis, revealed that, by reducing the expression of aromatase-mRNA with Letrozole, an aromatase inhibitor, size and volume of peritoneal lesions were diminished [50], suggesting a correlation between estrogen restriction and endometriosis lesion shrinking. In addition, progesterone antagonists (RU-486—mifepristone and ZK 98.299—onapristone) that block estrogen effects on endometrium and cause endometrial atrophy by suppressing proliferation were shown to be effective at both the reduction of the lesions and control of clinical symptoms. These studies initially conducted in non-human primate models reaffirmed the use of labeled drugs on endometriosis treatment by demonstrating how those therapies could be effective at controlling clinical symptoms [51]. However, none of the recent studies carried on these primates to test new drugs for endometriosis was found in the biomedical literature, possibly due to the prevalent culture of avoiding publication of negative results [52, 53]. Therefore, there is the possibility that non-human primate models are failing to reveal novel treatment strategies, since there has not been real progress in drug (label or off-label) management of endometriosis. The available treatments and/or surgery are often insufficient to eliminate the disease and to prevent its recurrence.

### Contribution of rodent models to endometriosis research

Because rodent models can be used for disease studies and can be genetically modified, they have been used to investigate endometriosis pathophysiology by means of several molecular techniques. The studies were focused mainly on finding possible new therapeutic targets and/or on improving existing ones [54], even though extrapolating data between species is difficult.

Isogenic mouse strains allowed mechanistic and regulatory approaches to investigation without the interference of the individual genetic variation existing in humans. These models offered insights into the effects of ectopic endometrial tissue growth [55], in vitro fertilization, embryo development and implantation, and oviduct transport [56]. Pain mechanisms [57], as well as inflammation [58] and organ adhesion [59] were also studied in mice. However, one of the major problems of using rodents in endometriosis research is that only superficial lesions can be induced in these animals and those are the simplest and perhaps the least clinically important types of lesion. No study to date has been able to model deep endometriotic lesions and perhaps this is one of the reasons for the lack of success of rodent models to generate applicable results to human endometriosis in the areas of pathophysiology and therapeutics.

Even though rodent models have provided information to endometriosis on inflammation [54], oxidative stress [60] and animal reproduction [61] there are still gaps in knowledge. Immune modulators [62] and antioxidant/oxidative stress compounds have also been studied in rodent models, but attempts to translate the results to humans did not lead to affective endometriosis therapies. It became clear that a number of women treated in clinical trials with hormonal therapies translated from results obtained in animal models of the disease did not respond to these treatments [63] needing surgical lesion excision to alleviate symptoms. It is important to mention that the current available hormonal therapies are not indicated for women trying to become pregnant because they interfere with ovulation. Because alternative therapies such as immune modulators have been shown to reduce lesion size in rodents [64] there was hope that they would also be effective in women and might improve fertility. However, this hypothesis was not confirmed in women with endometriosis.

The rodent model requires the surgical induction of endometriosis, usually done by transplanting endometrial tissue or cells into the animal’s abdominal cavity. This procedure leads to superficial endometriotic lesions, a model that basically favors the study of inflammatory processes caused by the implanted lesions instead of those caused by endometriosis itself. Recurrent studies in rodent models indicating putative therapeutic molecules, which eventually fail to be active in humans, are common in research on endometriosis, but also on other human diseases. One particular example is the treatment with resveratrol. In the mouse endometriosis-induced model this natural phenol showed promising results by reducing lesion size, inhibiting angiogenesis and inflammation in several overlapping studies [65–67]; however, when tested in affected women in a trial (ClinicalTrials.gov Identifier: NCT02475564) the results were disappointing.

Recently, a mouse model that mimics endometrial shedding similar to human menstruation was created. It is claimed to provide a closer model to human reproductive physiology. That model still requires more in-depth validation as it still presents limitations such as variation in the endometrial response, and in the duration and quantity of the hormonal stimulus [54, 68].

### Contribution of animal models to a breakthrough in endometriosis treatments: from disease experimental models to clinical trials

While there is exponential growth in the number of preclinical endometriosis studies, the translation of findings obtained by studies on animal models into clinical trials has been poor. A recent search in PubMed using a simple searching strategy “endometriosis and experimental model” retrieved 1230 published articles in February of 2019 compared to 469 in 2009, which amounts to a 150% increase. In fact, no novel therapeutic compounds for endometriosis have been proposed and there is only a small number of published clinical studies on drug development for endometriosis. This is an indication that the understanding of the disease and of its processes is still scarce [52, 69]. A fact that also reveals the failure of several attempts to translate experimental investigation results to the clinical sphere is evidenced by the number of collapsed phase II or III trials [28]. Again, most studies producing negative results and/or with conflict of interest are not published [52], a situation that leads to analytical bias and affects the perception of the existing state of research on endometriosis.

The high rate of potential drug therapies that fail at efficacy testing protocols in humans (Table 1) is noteworthy. Even when previous animal studies had shown that a treatment would be effective and safe, the subsequent failure rate in humans was higher than 80%. This situation suggests that preclinical animal studies are either not translatable at all or are not being accurately designed; therefore, studies of this type may be considered poor predictors of therapeutic efficacy of novel drugs in patients [70]. Although similar failure percentages are reported in drug discovery studies for other diseases, it brings into debate, in the case of endometriosis, the lack of reproducibility and translatability of animal studies to humans. The results stemming from animal research in the field of drug development would be a contributing factor to that observed failure when tested in human trials, raising the question of the quality of the results [71, 72].

**Table 1:** Number and characteristics of registered Clinical Trials on endometriosis

Clinical Trials Gov Identifier	StudyTitle	Phase	Conditions	Interventions	Aim	Comments	Conclusion
NCT01968694	Effects of Intravenous Lidocaine on Endometriosis Pain	Not applicable	Endometriosis	Drug: IV Lidocaine Drug placebo: IV diphenhydramine	Reduce pain	–	No statistical analysis provided
NCT00902746	Efficacy and Safety, Long-Term Study of NPC-01 to Treat Dysmenorrhea Associated With Endometriosis	3	Dysmenorrhea	Drug: NPC-01 (Norethisterone and Ethinyl Estradiol)	Reduce pain	Single Group Assignment (no placebo)	No statistical analysis provided
NCT02475564 NCT01620528 NCT01931670 NCT02143713 NCT01760954	Resveratrol for Pain Due to Endometriosis A Clinical Study to Evaluate the Safety and Efficacy of Elagolix in Subjects With Moderate to Severe Endometriosis-Associated Pain (ELARIS EM-I) A Global Phase 3 Study to Evaluate the Safety and Efficacy of Elagolix in Subjects With Moderate to Severe Endometriosis-Associated Pain (ELARIS EM-II) Global Study to Evaluate the Long-Term Safety and Efficacy of Elagolix in Women With Moderate to Severe Endometriosis-associated Pain Study to Evaluate the Long-Term Safety and Efficacy of Elagolix in Adults With Moderate to Severe Endometriosis-Associated Pain	4 3 3 3 3	Endometriosis Endometriosis Endometriosis Endometriosis Endometriosis	Drug: Placebo Drug: Resveratrol Drug: Placebo Drug: Elagolix Drug: Placebo Drug: Elagolix Drug: Placebo Drug: Elagolix Drug: Placebo Drug: Elagolix	Reduce pain Safety and efficacy + Reduce pain Safety and efficacy + Reduce pain Safety and efficacy + Reduce pain Safety and efficacy + Reduce pain	Authors conclude that a longer follow-up, for instance 6 months, may impact the results Participants who completed the 6-month treatment period in the pivotal Study M12-671 (NCT01931670) could enter this extension study Participants who completed the 6 month treatment period in the pivotal study M12-665 (NCT01620528). The study consists of 2 periods: a 6 month treatment period and a post treatment follow-up period of up to 12 months.	No difference in pain score, CA-125 and prolactin serum levels The use of Elagolix reduced pain and had adverse effects not yet analyzed by authors The use of Elagolix reduced pain and had adverse effects not yet analyzed by authors The use of Elagolix reduced pain and had adverse effects not yet analyzed by authors The use of Elagolix reduced pain and had adverse effects not yet analyzed by authors
NCT00973973	Efficacy and Safety Study of Elagolix in Women With Endometriosis	2	Endometriosis	Drug: Placebo Drug: Elagolix	Evaluate effects on endometriosis related pelvic pain and its safety	All participants still enrolled in the study received 150 mg elagolix once daily	The use of Elagolix reduced pain and had adverse effects not yet analyzed by authors
NCT00619866	An Efficacy and Safety Study of Elagolix (NBI-56418) in Women With Endometriosis	2	Endometriosis	Drug: Placebo Drug: Elagolix	Safety and efficacyand to see the effect, if any, on bone mineral density.		Only Elagolix at 8 week treatment had a statistical difference from the placebo group. Adverse effects and bone mineral density were not yet analyzed by authors
NCT00797225	Efficacy and Safety Study of Elagolix Versus Placebo or Leuprorelin Acetate in Endometriosis	2	Endometriosis	Drug:Leuprorelin Drug: Elagolix Drug: Placebo	Compare drugs safety and beneficial effects of elagolix	Leuprorelin is an approved endometriosis therapy	Elagolix treatment and Leuprorelin had a statistical difference from the placebo group. Adverse effects and bone mineral density were not yet analyzed by authors
NCT01791413	Effect of Pre-operative Depo Medroxyprogesterone Acetate on Serum Anti-mullerian Hormone Level After Laparoscopic Ovarian Cystectomy of Endometriomas	1 and 2	Endometriosis	Drug: Placebo Drug: depot medroxyprogesterone acetate	Ovarian reserve changes after preoperative medication	Ovarian endometrioma	No statistical analysis provided
NCT01269125	GnRH-a and Pregnancy Rate in In Vitro Fertilization (IVF) Cycles	Not applicable	Endometriosis Infertility	Drug: Leuprolide Procedure: IVF	Improve the oocyte quality and the fertility	Measured clinical pregnancy rate, embryo quality, fertilization rate, follicular fluid’s TNF-a concentration	No difference between groups
NCT01682642	The Influence of Adjuvant Medical Treatment of Peritoneal Endometriosis on the Outcome of IVF. A Prospective Randomized Analysis	4	Infertility Endometriosis	Drug: Zoladex	Impact of treatment prior to IVF on pregnancy rates	Number of Metaphase II Cells, Pregnancy Rate, Good Embryo Quality, Number of Pro Nuclear Cell (2PN), Number of Cryopreserved Embryos and Total Follicle Stimulating Hormone (FSH) Dose	No statistical analysis
NCT00474851	The Effect of Hormonal Add-Back Therapy in Adolescents Treated With a GnRH Agonist for Endometriosis: A Randomized Trial	2	Endometriosis	Drug: Norethindrone acetate + estrogens Drug: norethindrone acetate + placebo	Maintain skeletal health and quality of life in adolescents	Bone Mineral Density, Total Body Bone Mineral Content (BMC),	Total Body Bone Mineral Content was higher on the intervention group
NCT01791413	Effect of Pre-operative Depo Medroxyprogesterone Acetate on Serum Anti-mullerian Hormone Level After Laparoscopic Ovarian Cystectomy of Endometriomas	1 and 2	Endometriosis	Drug: depotmedroxyprogesteroneacetate	Evaluate ovarian reserve	Percentage changes of Serum Anti-Mullerian Hormone (AMH) at 2-week and 3-month post surgery	No statistical analysis
NCT01190475	BGS649 Monotherapy in Moderate to Severe Endometriosis Patients	2	Endometriosis	Drug: BGS649 Drug: Placebo	Assess the safety and tolerability	Proportion of patients who develop 2 or more follicles with diameter 16 mm or larger	No statistical analysis
NCT02203331	Bay98-7196, Dose Finding/POC Study	2	Endometriosis	Drug: Placebo Drug: Levonorgestrel Drug: Anastrozole Drug: Lupron/Leuprolide acetate	Assess efficacy and safety	Different Dose Combinations	No difference between groups
NCT01294371	Observational Program to Assess Routine Use of Add-back Therapy in Patients With Endometriosis in Russian Federation, Planned for 6-month Course of Lucrin Depot^®^ (Leuprorelin)	Not applicable	Genital Endometriosis	Non-interventional, observational study	Assess rates of administration of add-back therapy in patients with endometriosis		No statistical analysis

All information was taken from US National Library of Medicine, ClinicalTrials.gov without imposing dates or limits

We propose a thought-provoking illustration of that scenario in Table 2. We surveyed PubMed without determining dates for published articles on endometriosis that used animals as experimental models for testing anti-inflammatory drugs. With the intent of comparing the outcomes of animal studies, a search on *clinicaltrials.gov* was performed for registered clinical trials of the same drugs used as human medication. Clearly, the number of ongoing or complete clinical trials testing drugs previously evaluated in animal models for the same disease is small. Out of 36 drugs which had been tested in animal studies of endometriosis, 32 were object of clinical trials not related to endometriosis, while only 4 were (or are being) tested in clinical trials as treatments for endometriosis, namely: Resveratrol (NCT02475564), Yiweining [73], Curcumin (NCT03016039) and Rosiglitazone (NCT00115661; NCT00121953). Two of these four trials are finished by now, one is not yet recruiting, and one was interrupted (Table 2).

**Table 2:** All published research on endometriosis and anti-inflammatory drugs done in animal models was found by searching PubMed database and the corresponding clinical trials until January 2019

Author, year	Main drug	Drug effect	Animal model (n)	Conclusion	Clinical Trial using the same drug (ClinicalTrials.gov Identifier)	ClinicalTrial status	New endometriosis research advance
Saltan et al. (2016) [146]	Viburnum opulus	Antimicrobial, antioxidant, hepatoprotective, hypogly-cemic, antinociceptive and anti-inflammatory	Rat (30)	Endometrioctic volume reduced; Peritoneal TNF-α, VEGF and IL-6 concentration reduced	The Efficacy of Viburnum Opulus 3X in the Treatment of Primary Dysmenorrhea (NCT02467543)	Completed	Endometriosis was an exclusion criteria
Bostanci et al. (2016) [127])	Aloe Vera	Antioxidant	Rat (24)	Endometrioctic volume reduced; Peritoneal fluid antioxidant levels raised	43 trials using Aloe Vera, none using it to treat endometriosis	–	–
Zhou et al. (2012) [152]	Salvia miltiorrhiza Bunge	Anti-inflammatory, antioxidant	Rat (40)	Reduced levels of CA-125, TNF-α and IL-18. Increased levels of Il-13	2 trials with Salvia miltiorrhiza Bunge, one for Polycystic Ovary Syndrome and the other for Pulmonary conditions. None for endometriosis	–	–
Neto et al. (2011) [142]	Uncariatomentosa	Anti-inflammatory, immunomodulatory, pro-apoptotic, anti- oxidant and contraceptive	Rat (40)	Contraception	Phase II Clinical Trial of UncariaTomentosa (Cat´s Claw) in Patients With Advanced Solid Tumors (NCT02045719)	Unknown	Not associated with endometriosis
Sun et al. (2011) [149]	FubaoDangguiJiao	Abortion prevention and regulate menstruation	Rat (40)	Endometrioctic volume reduced	No clinicaltrials	–	–
Neto et al. (2011) [143]	Uncariatomentosa	Anti-inflammatory, immunomodulatory, pro-apoptotic, anti- oxidant and contraceptive	Rat (25)	Endometrioctic volume reduced	Phase II Clinical Trial of UncariaTomentosa (Cat´s Claw) in Patients With Advanced Solid Tumors (NCT02045719)	Unknown	Not associated with endometriosis
Qu et al. (2005) [145]	Yiweining	Anti-inflammatory	Rat (50)	Reduced serum levels of TNF-α, IL-6, and IL-8	Comparative study on the efficacy of Yiweining and Gestrinone for post-operational treatment of stage III endometriosis	Completed	Yiweining suppressed post-operational relapse and dissemination of endometriosis III
Xiao et al. (2002) [151]	Tripterygium Wilfordiipolyglycoside	Anti-inflammatory, immune modulation, antiproliferative, and proapoptotic	Rabbit (22)	Endometriotic volume and antiendometrial antibody reduced; serum FSH and LH levels decreased	6 clinical trials found on Pubmed. Noneendometriosisrelated	–	Its clinical use is limited duo its severe toxicity
Chen et al. (2010) [128])	15-epi-lipoxin A4	Anti-inflammatory	Mouse (45)	Supression of lesion growth	14 trials, none using for endometriosis research	–	–
Machado et al. (2010) [139]	cyclooxygenase-2 inhibitor	Inhibitor of the enzyme responsible for the production of prostaglandins which are one of the responsibles for inflammation	Rat (20)	Supression of lesion growth	682 trials, none using cyclooxygenase-2 inhibitor to treat endometriosis	–	–
Nenicu et al. (2017) [141]	1-Telmisartan and 2-parecoxib (cyclooxygenase-2 inhibitor)	1-Anti-hypertensive; 2-Inhibitor of the enzyme responsible for the production of prostaglandins which are one of the responsibles for inflammation	Mouse (42)	Supression of lesion formation and growth	282 and 47 trials, none using Telmisartan nor parecoxib to treat endometriosis respectively	–	–
Jana et al. (2012) [133])	Curcumin	Anti-oxidant and anti-inflammatory	Mouse (48)	Supression of lesion growth	Curcumin Supplementation for Gynecological Diseases (NCT03016039)	Not yet recruting	Curcumin was used to trattubo ovarian abcess, endometritis, wound infection.
Matsuzaki et al. (2004) [140]	cyclooxygenase-2 inhibitor	Inhibitor of the enzyme responsible for the production of prostaglandins which are one of the responsibles for inflammation	Rat (74)	Supression of lesion growth	682 trials, none using cyclooxygenase-2 inhibitor to treat endometriosis	–	–
Laux-Biehlmann et al. (2016) [138]	1-celecoxib (cyclooxygenase-2 inhibitor); 2-antinociceptive (Nav1.8 blocker A-803467)	1-Inhibitor of the enzyme responsible for the production of prostaglandins which are one of the responsibles for inflammation; 2-induce analgesia in inflammatory pain models	Mouse (number of animals was not described)	1-Decrease in percentage time in the standing position, associated with pain.	1. 496 trials using celecoxib, none for endometriosis; 2- 55 trials for antinociceptive none with endometriosis; 2-1 study using sodium channel blocker but not endometriosis related	–	–
Elmali et al. (2002) [130])	Caffeic acid phenethyl ester	Anti-inflammatory	Rat (30)	Decrease in osxidative stress parameters	1 clinical trial (NCT02744703) but not endometriosis related	–	–
Barretto et al. (2016) [125])	Acetylsalicylic acid	Cytolytic and antineoplastic	Rabbit (40)	Partial supression of lesion growth	1754 trails, none for endometriosis treatment	–	–
Abbas et al. (2013) [123])	β-Caryophyllene	Anti-inflammatory	Rat (27)	Supression of lesion growth without interfering with fertility	1 trial (NCT03152578) but not endometriosis related	–	–
Kizilay et al. (2017) [136]	1- Curcumin; 2-Deferoxamine	1-Anti-oxidant and anti-inflammatory; 2-Iron-chelating	Rat (30)	Supression of lesion growth	58 trials using Deferoxamine, but none for endometriosis treatment	–	–
Kiykac Altinbas et al. (2015) [135])	Montelukast	Anti-inflammatory	Rat (33)	Supression of lesion growth	287 trails using Montelukast (also searched for Singulair and MK 0476), but none for endometriosis research	–	–
Bayoglu Tekin et al. (2015) [126])	Resveratrol	Anti-angiogenic, antioxidant, anti-inflammatory	Rat (40)	Reduction on Anti-angiogenic, antioxidant, anti-inflammatory parameters	Resveratrol for Pain Due to Endometriosis (ResvEndo) (NCT02475564)	Completed (phase 4)	No difference between groups regarding pain and serum CA125 and Prolactin Levels reduction after 42 days of medication. 22 patients per group.
Hull et al. (2005) [132]	Nimesulide (COX-2 inhibitor)	Anti-angiogenic, antioxidant, anti-inflammatory	Mouse (30)	No supression of lesion growth	17 trails, none for endometriosis treatment	–	–
Efstathiou et al. (2005) [129])	1-Aspirin; 2-Celecoxib; 3-Ibuprofen; 4-Indomethacin; 5-Naproxen; 6-Sulindac; 7-Rofecoxib	Anti-angiogenic, antioxidant, anti-inflammatory	Mouse (105)	Celecoxib and indomethacin were most efficacious to supress lesion growth and aspirin had no effect	Ibuprofen is only used in 2 clinical trials but as rescue medication, not for testing (NCT02437175; NCT01942122)	–	–
Takai et al. (2013) [150]	Parthenolide	Anti-cancer and anti-inflammatory	Mouse (30)	Inhibited endometriosis like lesion formation	4 trials, none for endometriosis propose	–	–
Kurt et al. (2015) [137]	Colchicine	Anti-inflammatory	Rat (16)	Supression of lesion formation and growth	127 trials, none to study endometriosis	–	–
Güney et al. (2008) [131]	Melatonin	Antioxidant and anti-inflammatory	Rat (25)	Supression of lesion formation and growth	441 trials using Melatonin, none using it to treat endometriosis	–	–
Kilico et al. (2014) [134])	Dexketoprofen trometamol (Cyclooxygenase-2 enzyme inhibitor)	Inhibitor of the enzyme responsible for the production of prostaglandins which are one of the responsibles for inflammation	Rat (60)	Supression of lesion formation and growth	18 trials using Dexketoprofen trometamol (also searched as Enantyum), none to treat endometriosis	–	–
Rudzitis-Auth et al. (2013) [65]	Resveratrol	Anti-angiogenic, antioxidant, anti-inflammatory	Mouse (20)	Suppression of new microvessels in lesions	Resveratrol for Pain Due to Endometriosis (ResvEndo) (NCT02475564)	Completed (phase 4)	No difference between groups regarding pain and serum CA125 and Prolactin Levels reduction after 42 days of medication. 22 patients per group.
Agostinis et al. 2015 [124])	1- N-acetyl cysteine; 2-alpha-lipoic acid; 3- bromelain	Antioxidant, anti-inflammatory	Mouse (16)	Supression of lesion formation and growth	No endometriosis related clinical trial with neither drug	–	–
Soylu Karapinar et al. (2017) [148]	Dexpanthenol	Antioxidant, anti-inflammatory	Rat (20)	Supression of lesion growth	26 trials using Dexpanthenol (or Panthenol and Bepanthen), but nnone for endometriosis treatment	–	–
Siqueira et al. (2011) [147]	Acetylsalicylic acid	Cytolytic and antineoplastic	Rabbit (40)	Supression of lesion growth	1754 trails, none for endometriosis treatment	–	–
Olivares et al. (2011) [144]	Celecoxib (Cyclooxygenase-2 enzyme inhibitor) and rosiglitazone	Anti-angiogenic, antioxidant, anti-inflammatory	Mouse (48)	Supression of lesion formation and growth	1-Use of Rosiglitazone in the Treatment of Endometriosis (NCT00115661); 2-ffect of Rosiglitazone on Peritoneal Cytokines in Women With Endometriosis (NCT00121953)	1-Terminated; 2-Withdrawn	–

That small number of clinical trials shows that previous animal testing for endometriosis-active drugs does not translate in clinical trials to verify their effects on patients. We searched the United States of America National Library of Medicine—*clinicaltrial.org* website and found 213 registered clinical trials relating to endometriosis drug testing (last search was performed January 2019), 105 were “Completed”, 36 were “Recruiting” or “Enrolling by invitation”, 28 were “Not yet recruiting”, “Active”, or with “Not recruiting status”, 20 were “Suspended”, “Terminated” or “Withdrawn” and 24 had “Unknown status”.

For all completed trials, only 19 were published and, of these, 18 aimed at finding novel therapeutic activities for specific endometriosis symptoms. All the investigated drugs had already been tested for other diseases and were approved by the FDA. Some of the tested drugs were specifically “labeled”, while others were “off-label” drugs used for endometriosis treatment (Table 1) [51]. Out of those 18 studies, seven aimed at pain reduction, four were focused on fertility improvement, one study aimed at improving life quality of adolescents and nine studies aimed at improving drug tolerance and safety (some trials had multiples aims). Interestingly, none of these drugs undergoing new therapeutic testing originated from preclinical studies using animal models.

Another important point that reveals the lack of comparability between clinical trials and animal studies is the fact that, while animal-based studies on experimental drugs have lesion reduction as measured positive outcome of the test, most clinical trials have as main outcome the reduction of disease symptoms (mainly pain and infertility, or fertility preservation).

Moreover, the endpoint of most animal model studies is based on lesion regression, as if it would be considered an important endpoint in endometriosis research. However, endometriosis clinical trials (Table 1), do not concern and do not look for lesion regression when studying a potential new drug. Curiously, lesion regression has never been chosen as an end-point of clinical studies. Clinical studies were aimed at finding ways to mitigate symptoms, which seems to be the utmost point to be addressed for endometriosis [8, 74, 75]. An International Consensus Workshop proposed a list of priorities for endometriosis research and under the “treatment and outcome” section, lesion regression was not mentioned at all, as opposed to diminishing symptoms [76]. According to Paolo Vercellini´s view, the treatment should not target the lesions but the clinical signs, as researchers should aim at providing patients comfort and address their main complaints [74] instead of masking advances with endpoints of questionable use for the progression of new drugs discovery. Thus, it is still debatable what would be the most adequate approach to study endometriosis. If endometriosis is seen as a chronic, yet incurable, but not a life-threatening disease, then mitigation or the end of symptoms seem to be reasonable approaches to improve quality of life for the patients. At least, this would be a goal until the etiology of endometriosis becomes clear and, in consequence, research will seek novel approaches.

## In vitro studies using patient-derived tissue and fluids, and in vivo human-based-models

Although experimental research on animals whose goal was lesion regression failed to produce translatable data to humans, human-based research approaches to endometriosis (including clinical trials, observation and association studies) are generating the most relevant publications in this field. Notably, the most cited original and currently used article for understanding and classifying endometriosis grades is the *Revised American Society for Reproductive Medicine classification of endometriosis: 1996.* This article reports clinicians’ observations of disease behavior by visual and histopathological analysis of biopsies [77]. In addition, when revising high-impact of endometriosis-narrative reviews [78], the bibliography cited by the reviewers focused mostly on in vitro patient-derived tissue and/or fluids and on human-based studies, the ones that were considered to advance our knowledge of endometriosis. It is worth noticing that among most highly cited papers on endometriosis, there are two human-based studies. One of these deals with the deleterious effect of continuous ovulatory cycles on endometriosis persistence [79] and the other revealed a mutation in the *ARID1A*in gene found in endometriosis-associated ovarian carcinomas [80]. Furthermore, a highly innovative target for the non-hormonal treatment of endometriosis, P2X3, which is thought to be associated with development and maintenance of chronic pelvic pain, was also identified without using animal models [81].

Highlights of advances brought about from human-based research approaches comprise: large-scale integrated genome-wide RNA sequencing [82]; endometriosis peritoneal fluid a potent oxidative fluid and modifier of miRNA expression profile in eutopic cells [83]; miRNA analysis associated with pelvic pain [84]; modulation of pain [85], anxiety and depression [86]; tissue-specific expression analysis of endometriosis tissue samples by laser capture microdissection (reviewed in [87]); genetic associations between endometriosis and obesity-related traits [88].

Genomic wide association studies (GWAS) have emerged more than a decade ago [89] as an important tool to study complex genetic diseases. Particularly for endometriosis, GWAS has helped to infer causality and uncover pathogenic mechanisms. It may be helpful in order to dissect all possible phenotypes of the disease, although this has not been broadly tested yet. To date, GWAS revealed more than 10 genomic regions associated with endometriosis, a condition that explains less than < 4% of inheritance [82, 90]. Some of these studies revealed, for instance, novel associations between endometriosis (and some specific phenotypes) with the Wnt Family Member 4 (WNT4) locus [91] and the mitogen-activated protein kinase (MAPK)-related pathway [90]. The gathered information has allowed new insights into the biological pathways that could be associated with the pathology of endometriosis, besides being indicative of new potential therapeutic targets.

## Contributions of in vitro models for endometriosis research

Biopsies are important cell sources for in vitro culture. In endometriosis research cells cultured on flat surfaces, also called two-dimensional (2D) cellular models, have generated hundreds of publications. Co-culture models are cell cultures in which two (or more) different cell types are cultured together, often separated by a fluid-permeable membrane. They are mostly used to assess the effect of one cell type on another cell and also to verify how cells interact [92]. These two previous models lack important features for cellular function feature such as a cellular physicochemical microenvironment, tissue-specific architecture, and blood flow perfusion. In the quest to create better models to observe cell to cell interaction, investigators have recently started to use three-dimensional (3D) models (sometimes called spheroid or organoid cultures) [93]. These 3D models aim at providing better phenotype and gene expression than 2D culture systems, opening the possibility to test interactions among different cells found in endometriotic lesions [94].

By means of in vitro conducted experiments, researchers have found elevated levels of cytokine and inflammatory mediators in the peritoneal cavity and phenotypic progesterone resistance, a condition seen in endometriosis patients [95]. Progesterone has anti-inflammatory activity and the results show a link between progesterone-resistance and chronic inflammatory states. Signaling by progesterone in endometrial cells induces suppression of NF-κB, which stands for nuclear factor kappa-light-chain-enhancer of the family of B proteins [96]. This suppression may lead to the establishment and maintenance of endometriosis implants because NF-κB has the ability to regulate a large array of genes involved in different processes of immune and inflammatory responses, thus controlling genes related to cell proliferation, adhesion, apoptosis, and inflammation [97].

These findings have emerged from in vitro observations and originated a whole new group of drugs for endometriosis treatment. One example are the progestins, a class of drugs that inhibit inflammatory pathways, angiogenesis and oxidative stress in endometriotic cells. These and other observations have opened up a new field, which may be critical for understanding mechanisms underlying the development, progression, variability and symptomatology of endometriosis.

In addition, a study with 2D cell cultures found that inhibition of IL-1β in eutopic endometrial stromal cell cultures could potently alter decidualization in vitro, suggesting a way to improve endometrial receptivity and pregnancy success in women with endometriosis [98]. Moreover, with the use of 2D culture of eutopic endometrial stromal cells, researchers found increased expression of a gene (*H19* gene) in endometriosis. This gene is responsible for tumor cell invasion and migration, and is regulated by estrogen and progesterone. The dysfunction of such a gene might act as an important factor for endometrial stromal cell invasion and migration, contributing to fibrous tissue formation [99].

Basic research studies using cell cultures from endometriosis patients shed light on cellular behavior and possibly new ways of managing cellular growth. Dienogest was shown to downregulate the expression of *CYP19A1* (aromatase gene), inflammatory and neuroangiogenesis in immortalized endometriotic epithelial cell lines [100], also in a spheroid cell culture system (3D system), dienogest inhibited mRNA prostagladin synthases, protein expression, and the nuclear factor-κB activation, which could contribute to therapeutic effect on endometriosis [101]. Recently, a double-blind phase 3 clinical trial showed the benefits of using dienogest for endometriosis treatment, by reducing endometriosis-associated pelvic pain [102], which could be also related to inhibition of prostaglandin mRNA synthases.

## Roadmaps for endometriosis research: perspectives on new approaches of endometriosis

The ultimate desirable goal of modeling endometriosis is to be able to develop a strong project, hypothesis-driven, and based on solid science. We envision that with the advance of high throughput genomics analysis, epigenomics, transcriptomics, proteomics and metabolomics in cell biology, these analyses will be carried out in endometriosis-derived tissue samples. There will be a need for an integrated guideline of all “*omics”* to facilitate the access to research data and to validate and increase their reproducibility and robustness rates. This should also facilitate both interpretation and functional use of such data [103]. Importantly, it is expected that these data will provide alternative strategies for diagnosis and target the treatment of symptoms. The high-dimensional gene expression data presented by Linda Giudice’s group [104] represent the first steps toward that approach, by putting forward a molecular classification of endometrium candidate-genes and offering a correlation with disease conditions and stage.

Besides inherent technical limitations such as difficulties to reach statistical power because of the large number of observations required, GWAS has recently allowed new insights into novel endometriosis-associated biological and pathogenetic pathways and giving rise to new hypotheses to understand that disease. For instance, a GWAS study suggested that the pathogenesis of advanced endometriosis is likely to be distinct from less severe disease [82]. This hypothesis is possible due to the integration of molecular sequencing/expression results with detailed clinical and demographic data. In fact, GWAS seems to have the potential to cover the complete spectrum of disease-related effects [82]. In endometriosis, no publication has yet explored the so-called phenomics (the set of phenotypes physical and biochemical traits by the organism due to genetic and environmental influences) approach, although this is a promising avenue [82]. Moreover, PhenoWAS, (Phenomics Wide Association Analysis) is an opposite way of looking at data in comparison to GWAS classical approach, and might serve as a powerful way to investigate endometriosis. PhenoWAS consists of investigating which diseases (many diseases at the same time) are associated with a given genetic variant [105]. This approach is only possible if a high-dimensional phenotypic dataset is available or very large and well phenotypically-characterized cohort is used.

Years ago, it would have been impossible to foresee the emergence of artificial intelligence (AI) models to advance our knowledge on disease. However, text analytics (text mining based on AI), high-throughput cell-based assays, automated microscopy-based high-content screening and convolutional neural network can facilitate the way we look at data. Text mining is a process of adapting a massive amount of unstructured information into significant interpretable data for research analysis, such as proteins, genes and other markers to eventually reach a set of findings relevant to endometriosis research [106]. AI can be applied to high-throughput cell-based assays’ data and to automated microscopy-based high-content screenings. Both approaches allow the parallel monitoring of multiple cell phenotypes, as well as the examination of cell shape changes under different conditions and the staining for surface and cytoplasmic molecules by fluorescence-labeled antibodies.

In endometriosis, AI and text mining were put to prove by unraveling endometriosis most important associated genes that were already published, besides discovering new ones, building a genome wide gene network [107]. This network, coupled with other AI applications, formed a list of 5 top genes to be studied in endometriosis and perhaps used as biomarkers for the disease. Another work implemented machine learning AI to find a relationship between endometriosis and other diseases, as for instance benign breast disease, cystitis and non-toxic goiter. Using a similarity matrix, the method evidenced other diseases that women with endometriosis had in common [108], showing that this would be a useful method for understanding how endometriosis works and how it could be associated with other disease and symptoms.

These techniques facilitate phenotype measurements of individual cells and analyses of heterogeneous responses, providing in-depth insights into biological processes [109]. Convolutional neural network (CNN), multi-scale convolutional neural network (MSCNN) and deep convolutional neural network (DCNN) are remarkable recent AI advances. Developing rapidly over the past 6 years, those approaches are capable of fast “learning” from captured images (by sensing pixel intensity values) or from input data. By teaching itself, these programs can automatically improve their analysis (machine learning) [110, 111]. In order to become the next-generation tool for the diagnosis of human disease, CNN in all its different forms, MSCNN and DCNN, are being tested and compared to diagnosis by clinicians, proving to be more accurate, sensitive and specific ways for the classification of some diseases such as lung cancer [112]. A potential contribution of such a system to clinical decision making and therapeutic management of endometriosis should help with earlier diagnosis. This may contribute to identify disease phenotypes and how they relate to clinical symptoms, thus offering the possibility of custom-tailored treatments according to phenotypes (based on symptoms, images or gene expression features) and genotypes (based on DNA sequencing). In addition, AI combined with bioinformatics can be useful for retrieving relevant data on novel targets or markers for endometriosis [113], as well as improving diagnosis, the understanding of gene pathways and protein networks.

Cell-culture has helped our understanding of gene and protein expression [114], the roles of hormones in endometriosis [115] and also the possibility to recreate early stages of endometriosis in a “Petri-dish” [116]. Three-dimensional models offer the opportunity to study aspects of endometrial stromal and epithelial cells communication and paracrine cross-talk between stromal and epithelial cells [117]. While endometrial and endometriotic cells can also be cultivated with biopolymer and pre-fabricated scaffolds, hydrogels and cell sheets, microfluidic devices like “organ-on-chips” are gaining space as new research models for endometriosis [118]. Organ-on-chips allows morphological and functional changes of cells to occur in a microfluidic device, with the aim of reproducing enough cellular functions that the model can be used to test therapeutic drugs and toxicity effects [119]. By creating an “endometrium-on-chip” to study the role of the perivascular stroma in the human endometrium, this method could indirectly offer information on in vivo mechanisms [120]. Moreover, the breakthrough of cultivating the reproductive tract “on-a-chip” [121] has offered hope to model cyclic hormonal effects in hormone-dependent diseases, such as endometriosis. Thus, endometriosis research can now count on several new approaches to improve our knowledge that is, yet, rather limited.

The lack of translation of results from animal to human has been highly disappointing. Situations like this have led many researchers to consider the use of animals to model human disease is a limited path concerning time, resources, and money. Perhaps a positive outcome of the failure in translating research focused on therapy from animal experiments to human trials is the recognition of the limitations of using animals for experimental purposes [35].

## Final remarks

While researchers working with rodent and other animal models of endometriosis investigate reduction of lesion size and of cell proliferation, apoptosis parameters, or protein and gene expression data, from the point of view of achieving or advancing therapy for human endometriosis these models have provided scanty results. Analysis of available reported publications shows that animal models hardly produce robust data to provide candidate drugs for clinical trials. Thus, it is questionable whether these models should constitute the main strategy for understanding endometriosis pathophysiology and for the development of new therapies.

Hardly new compounds will emerge for the treatment of endometriosis based on therapeutic interventions in animal models. It is important to note that although the use of animals presents handling and physiological disadvantages, regulatory agencies (i.e., FDA United States and ANVISA Brazil) still require small and large animals for new drug testing, particularly for efficacy, toxicology and safety analysis. A topic of discussion by the scientific community, industries and regulatory agencies are alternatives to in vivo testing of efficacy, toxicology and safety of new drugs, and the real value of in vivo tests. In vitro and in silico tests have been established and are under validation, particularly for efficacy studies, instead of using naturally or induced animal models. Advances in in vitro modeling technologies, as for instance 3D blood vessels printing [122], are promising new platforms for the understanding of angiogenesis and vasculogenesis physiology.

## Conclusion

Finally, we envision personalized treatments that would utilize complex decision algorithms (text mining, neural networks, etc.) capable of integrating genomics, transcriptomics, epigenomics, proteomics, microbiome, exposures, behaviors, informatics and clinical/phonotypical data both cross-sectionally and throughout the patients’ lifetime. In our view, health regulatory agencies should question the currently required preclinical data from non-translatable animal models, and instead more multicenter, randomized controlled trials should be performed to test different endometriosis treatment options against defined outcome measures, such as relief of pain and/or infertility. This will bring back quality of life to endometriosis-suffering women.

## Data Availability

All data generated or analysed during this study are included in this published article [and its supplementary information files].

## Abbreviations

CNN: Convolutional neural network
DCNN: Deep convolutional neural network
GWAS: Genomic wide association studies
MSCNN: Multi-scale convolutional neural network
SCHEER: Scientific Committee on Health Environmental and Emerging Risks
3D: Three-dimensional
